# Treatment Outcome of Epileptic Patients Receiving Antiepileptic Drugs in Ethiopia: A Systematic Review and Meta-Analysis

**DOI:** 10.1155/2021/5586041

**Published:** 2021-05-13

**Authors:** Taklo Simeneh Yazie, Belayneh Kefale, Mulugeta Molla

**Affiliations:** ^1^Pharmacology and Toxicology Unit, Department of Pharmacy, College of Health Sciences, Debre Tabor University, P.O. Box 272, Debre Tabor, Amhara, Ethiopia; ^2^Clinical Pharmacy Unit and Research Team, Department of Pharmacy, College of Health Sciences, Debre Tabor University, P.O. Box 272, Debre Tabor, Amhara, Ethiopia

## Abstract

**Background:**

The prevalence and incidence rate of epilepsy were found to be higher in low- and middle-income countries. Uncontrolled epilepsy has a high risk of disability, stigma, discrimination, human rights violations, and premature death. The available studies of controlled seizure in Ethiopia have showed inconsistent results which calls for systematic review and meta-analysis. Therefore, this review intended to show the pooled prevalence of controlled seizure among people with epilepsy receiving antiepileptic drugs at outpatient department.

**Methods:**

A systematic literature search was conducted using PubMed/Medline, Science Direct, PsycINFO, Hinnarri databases, and Google Scholar for grey literatures. Data were extracted with structured format prepared using Microsoft Excel and exported to Stata/MP 16.0 software for analyses. The *I*^2^ test was used to check the heterogeneity between primary studies with a corresponding 95% confidence interval (CI).

**Results:**

A total of 23 primary studies were included in the review showing the pooled prevalence of controlled seizure to be 46% (95% CI: 35, 56). A subgroup analysis of the primary studies showed a considerable variation in magnitude of seizure freedom by study regions, age groups, and seizure-free period. The highest prevalence was found in Addis Ababa 52% (95% CI: 29, 75), pediatric patients 77% (95% CI: 71, 83), and a seizure-free period of less than six months 58% (95% CI: 32, 83). On the other hand, the lowest prevalence of controlled seizure was found in Tigray 27% (95% CI: 11, 65), adult patients 43% (95% CI: 32, 54), and a seizure-free period of six or more 41% (95% CI: 32, 51). Higher frequency of seizure before treatment (2.23, 95% CI: 1.15, 3.31) and medication nonadherence (2.7, 95% CI: 1.25, 4.15) had statistically significant association with uncontrolled seizure.

**Conclusion:**

In this review, the prevalence of controlled seizure was found to be low. This warrants that clinicians should give more focus to epileptic patients regarding monitoring and evaluation of treatment outcome of epilepsy and factors that affect seizure control in routine clinical services. The use of standardized definition of controlled seizure, designing strategies to identify pharmacoresistant epilepsy and its treatment, and increasing medication adherence are recommended in Ethiopia. The review protocol has been registered with PROSPERO registration number CRD42021215302.

## 1. Introduction

Epilepsy is a neurological disorder in which brain activity becomes abnormal, causing seizures or periods of unusual behavior, sensations, and sometimes loss of consciousness [1]. The International League Against Epilepsy (ILAE) proposed that epilepsy is considered as a disease of the brain defined either by the occurrence of at least two unprovoked seizures greater than 24 hours apart or one unprovoked seizure and a probability of further seizures similar to the general recurrence risk (at least 60%) after two unprovoked seizures, occurring over the next 10 years or diagnosis of an epilepsy syndrome [2].

Epilepsy accounts for a significant proportion of the world's burden of disease, affecting around 50 million people worldwide [1]. The active annual period prevalence, lifetime prevalence, and incidence rate of epilepsy were higher in low- to middle-income countries (LMIC) than high-income countries (HIC). This difference can be explained by the difference in prevalence of risk factors, treatment gap, and methodological issues such as definition of cases [3]. Nearly 80% of people with epilepsy live in low- and middle-income countries (LMIC), where treatment gaps exceed 75% in most low-income countries and 50% in most middle-income countries [1]. In Ethiopia, the prevalence of epilepsy was 5.2/1000 inhabitants at risk, 5.8 for males and 4.6 for females [4].

Epilepsy has a high risk of disability, stigma, discrimination, human rights violations, and premature death. Up to 70% of people with epilepsy could become seizure-free with appropriate diagnosis and use of cost-effective and commonly available antiseizure medicines. This seizure-free status can lead people with epilepsy to continue or return to a full and productive life [1]. For patients with medication-resistant convulsive epilepsy, newer antiepileptic drugs like lamotrigine, levetiracetam, and topiramate should be offered as add-on therapy [5]. In Ethiopia, there was discrepancy on the prevalence of controlled seizure among patients receiving antiepileptic drugs that ranges from 8% to 93.8% [6, 7]. Poor treatment outcome can be due to nonadherence to antiepileptic drugs [8, 9], and most patients rely on traditional treatments of epilepsy such as local herbs, holy water, and amulets [4]. The discrepancy of results between individual studies and the absence of systematic review on treatment outcome of epilepsy in Ethiopia warrants the need of systematic review which shows the pooled prevalence of controlled seizure among patients receiving antiepileptic drugs.

## 2. Methods

### 2.1. Reporting and Protocol Registration

This review followed the preferred reporting items for systematic reviews and meta-analysis guideline (PRISMA-P) protocol [10]. The review protocol has been registered in the international prospective register of systematic reviews (PROSPERO) with registration number of “CRD42021215302.”

### 2.2. Data Sources and Search Strategy

Search of both published and unpublished primary articles related to treatment outcome of epileptic patients attending outpatient department was conducted. A systematic literature search was conducted using PubMed/Medline, Science Direct, PsycINFO, Hinnarri databases, and Google Scholar for grey literatures. We also searched literatures using the direct websites of local (Ethiopian) journals. The key terms used to retrieve primary articles were treatment outcome OR patient-relevant outcome OR clinical effectiveness OR treatment effectiveness OR rehabilitation outcome OR disease-free survival OR progression-free survival OR treatment failure OR seizure control AND antiepileptic drugs OR anticonvulsant drugs OR antiseizure drugs AND epilepsy OR epileptic Syndromes OR seizure AND Ethiopia. All important literatures available from January 1, 2000, to November 15, 2020, having reports of prevalence of treatment outcome were included in this systematic review and meta-analysis.

### 2.3. Eligibility Criteria

The overall identified studies were exported to the EndNote citation manager to avoid duplications and then assessed for their eligibility to be included in this systematic review and meta-analysis using a prepared Microsoft Excel assessment format.

#### 2.3.1. Inclusion Criteria

*(1) Study Area*. Research articles conducted in Ethiopia were included in this review.

*(2) Study Design*. Observational studies (cross-sectional, case-control, and cohort studies) with original data reporting the prevalence of controlled seizure were considered as eligible to be included in this review.

*(3) Language*. Literatures written in the English language or had additional English version were included.

*(4) Population*. Studies conducted among adult and pediatric patients receiving antiepileptic treatment were included.

*(5) Publication Issue*. Both published and grey literatures available from January 1, 2000, to November 15, 2020, were included.

#### 2.3.2. Exclusion Criteria

Articles with insufficient information, records with missing outcome of interest, findings from personal opinions, editorial reports, and letters to the editors, case reports and series, systematic reviews, and qualitative studies were excluded.

### 2.4. Study Selection

The two authors (TS and BK) independently evaluated the eligibility of primary studies to be included in this review using the PRISMA guideline [10]. First, duplication of articles was avoided from the overall identified studies using EndNote citation manager. Then, articles were evaluated by reading their titles and abstracts. In the title and abstract evaluation, studies reporting at least one of the following (antiepileptic drug, antiepileptic medication, treatment outcome, seizure control, epilepsy, or seizure) were considered for further evaluation by full-text reading. After reading the full texts of selected articles, studies fulfilling the eligibility criteria were included in this systematic review and meta-analysis. Disagreements between the two assessors were solved by reevaluating the eligibility by both authors together.

### 2.5. Outcome Measurement

Outcomes of this systematic review and meta-analysis are the pooled prevalence of controlled seizure among people with epilepsy in Ethiopia. Controlled seizure was measured from the direct reports of the primary studies. We also measured controlled seizure from studies in which seizure control was measured as an explanatory variable of other outcome variables among people attending epilepsy outpatient treatment in Ethiopia. In the primary studies, seizure-free period was assessed by arranging outpatient clinic scheduled visits.

### 2.6. Quality Assessment

Both authors independently evaluated the overall qualities of the primary articles using the Newcastle-Ottawa Scale for cross-sectional and cohort studies quality assessment tool [11, 12]. The tool had different indicators consisting of three main parts. The first part of the tool had five components used to assess the methodological quality of each study. The second section examines the comparability of primary studies. The last part also measures the quality of the original articles with respect to their statistical analysis and interpretation. Any disagreements between two assessors were negotiated through discussion and by taking the average score of the two different assessment results. Articles fulfilling 6 and above points of quality assessment criteria score were included in this review.

### 2.7. Data Extraction

Data was extracted by using a standardized data abstraction form prepared in Microsoft Excel by reviewers (BK and TS). The authors extracted important data related to the study characteristics (the region and the study area in Ethiopia, title, the first author, and the year of study and publication), the study design, the population characteristics, the sample size, primary and secondary variables/outcomes of interest, and the main outcomes of interest (the effect size data, including the prevalence of controlled seizure and the associated factors of controlled seizure). Disagreements between the two assessors were solved by reevaluating the eligibility by both authors together. The event rate (proportion) was calculated, and standard error of Logit event rate was added using Comprehensive Meta-Analysis (CMA) version 3 software.

### 2.8. Statistical Procedures

The extracted data were imported from the Microsoft Excel data extraction format to Stata Version 16.0 software for analysis. The standard error for the prevalence of controlled seizure was calculated using the binomial distribution formula for each original article. We checked the heterogeneity of primary studies using *I*^2^ test. Based on the test result, a random-effects meta-analysis model was used to estimate DerSimonian and Laird's pooled effect of controlled seizure. In addition, subgroup analysis was performed based on screening tools used to measure controlled seizure to minimize the random variations between the point estimates of the primary studies. Potential publication bias had also been examined through visual assessment of the funnel plot and Egger's test statistics at 5% significance level.

## 3. Results

### 3.1. Search Results

**Figure 1:**
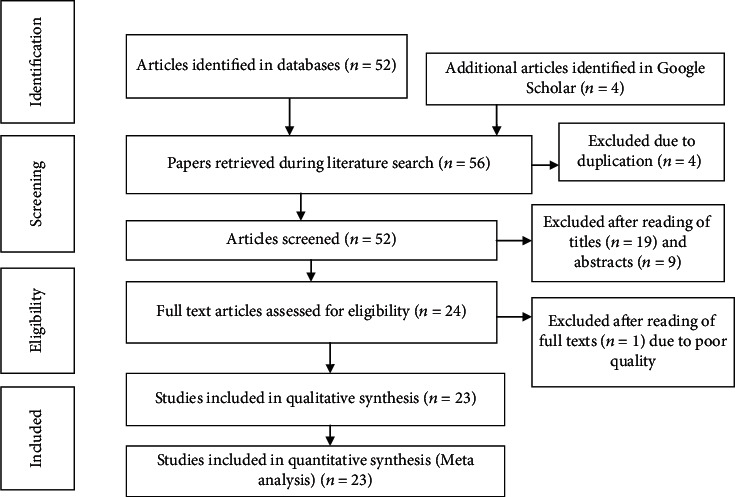
Flowchart showing the selection process of primary studies.

The database and manual searches of literatures yielded a total of 56 primary articles. Four articles were removed due to duplication. The remaining 52 articles were evaluated by reading their title and abstracts. During title and abstract evaluation, 28 articles were excluded and 24 articles were selected for further evaluation by reading their full texts. After full-text reading, 23 articles fulfilled the inclusion criteria and were included in the systematic review and meta-analysis (Figure 1).

### 3.2. Characteristics of the Included Primary Studies

Twenty-three primary studies were included in the present systematic review and meta-analysis. These studies were conducted in different parts of Ethiopia (from Amhara seven primary articles [7, 13–18], Oromiya eight [8, 9, 19–24], Addis Ababa four [25–28], Tigray two [9, 29], and Southern Nation, Nationalities, and People two articles) [30, 31]. The sample size of the included primary studies was considerably variable ranging from 121 to 415 participants. Regarding the seizure-free period, two studies assessed seizure freedom over one month [6, 21], one study over two months [17], two studies over three months [7, 14, 25], and the remaining studies assessed seizure freedom over at least six months. Concerning the age groups, one study included only pediatric patients [7], two studies included all age group patients [13, 19], and the remaining studies included only adult patients [6, 8, 9, 14–18, 20–31]. Regarding the antiepileptic drugs, most of the patients were treated with monotherapy and the commonly used drugs were phenobarbitone, phenytoin, carbamazepine, and valproic acid [6–8, 17, 20, 26]. The subgroup analysis was done in this systematic review and meta-analysis based on the screening tools that have been used by the primary studies to measure prevalence of participants with seizure freedom.

The Newcastle-Ottawa Scale quality assessment tool was used to assess the quality of primary studies, and the quality score of studies was acceptable with score of 6 and above points from a total of 9 or 10. In this review, the pooled prevalence of treatment outcome of participants receiving antiepileptic drugs was calculated from a total of 7255 participants attending follow-up service for epilepsy in Ethiopia using a total of 23 primary studies (Table 1).

### 3.3. Seizure Freedom among Epileptic Patients Receiving Antiepileptic Drugs

**Figure 2:**
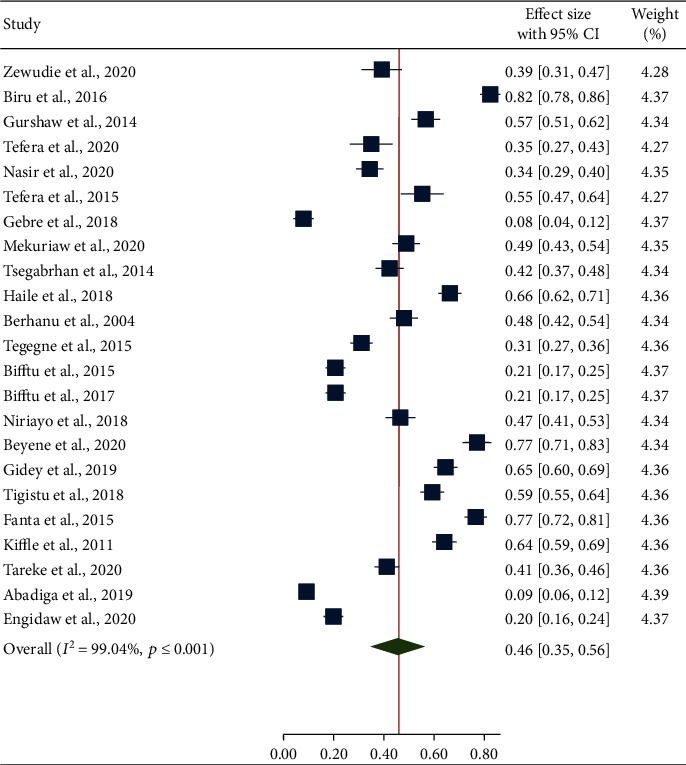
Forest plot illustrating the pooled prevalence of seizure freedom of 23 studies.

The prevalence of seizure freedom was varied considerably across reports of the primary studies in Ethiopia. The smallest prevalence of seizure freedom was 8% as reported by the study conducted at Ayder Comprehensive Specialized Hospital and Mekele Hospital [6]. The highest prevalence of seizure freedom (82.4%) was reported by the study conducted at Gondar University Teaching Hospital [14]. The overall pooled prevalence of seizure freedom was found to be 46% (95% CI: 35-56) among all age group patients attending antiepileptic treatment in Ethiopia (Figure 2).

### 3.4. Subgroup Analysis of the Prevalence of Seizure Freedom

**Figure 3:**
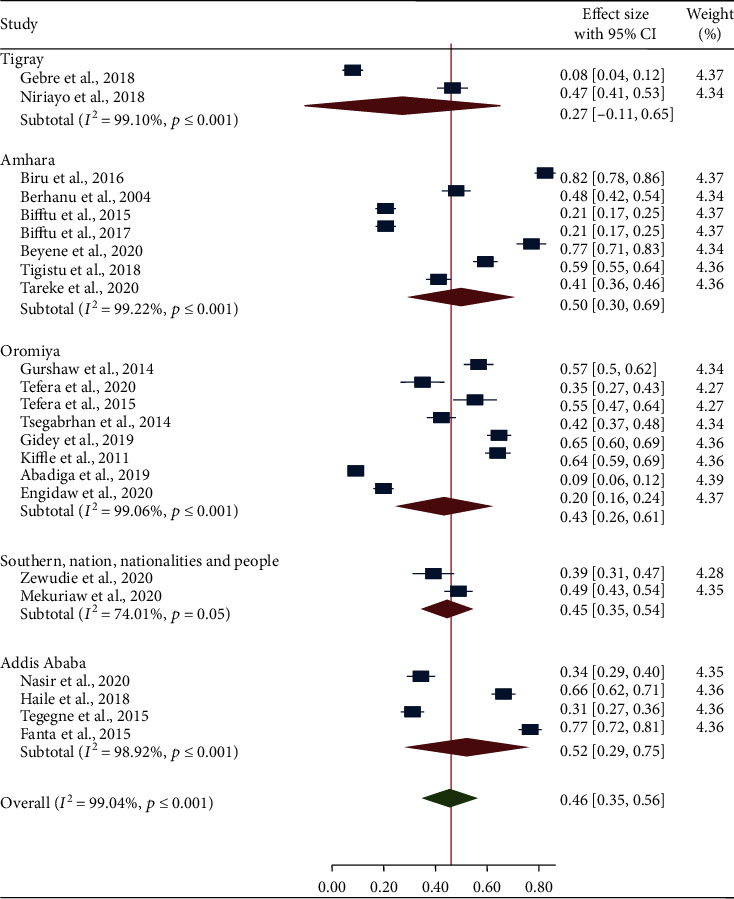
Forest plot depicting subgroup analysis of the pooled prevalence of seizure freedom by study regions.

**Figure 4:**
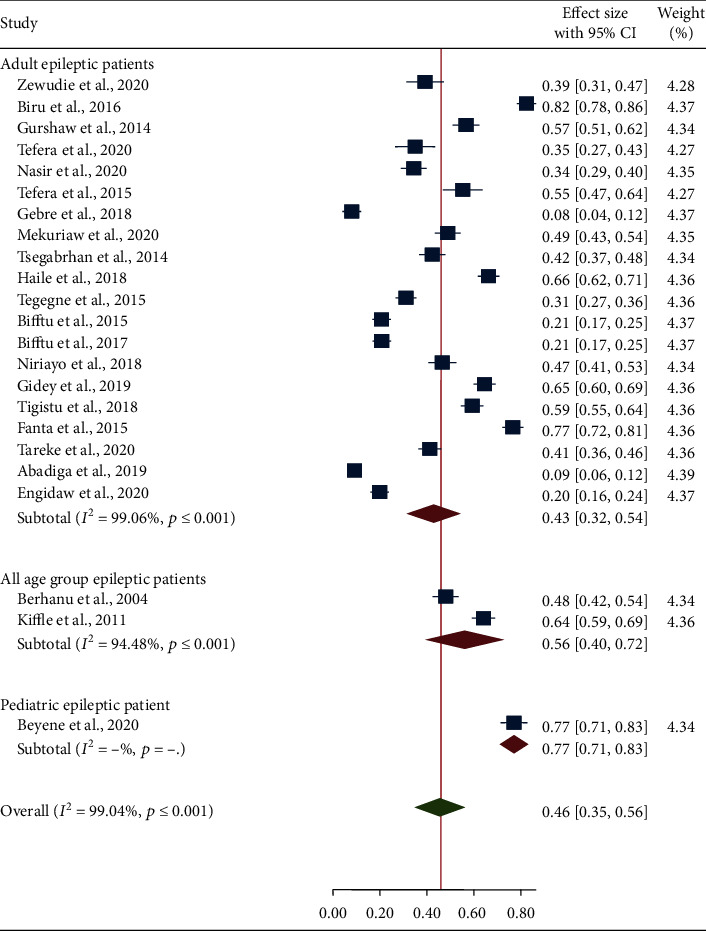
Forest plot depicting subgroup analysis of seizure freedom by age groups.

**Figure 5:**
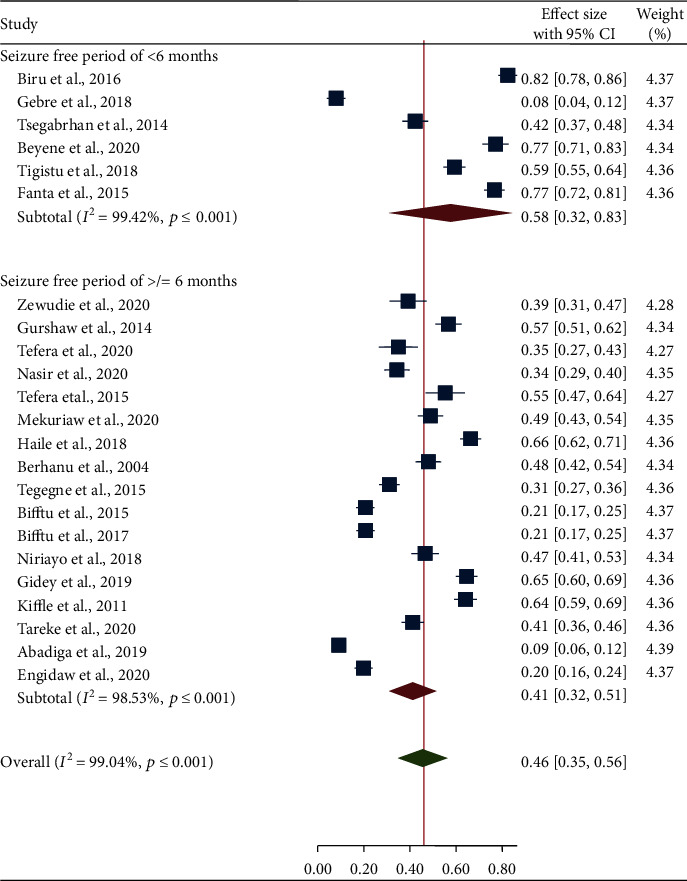
Forest plot illustrating subgroup analysis of seizure freedom by seizure-free period.

A subgroup analysis of the primary studies showed a considerable variation in magnitude of seizure freedom by study regions, age groups, and seizure-free period. In subgroup analysis of seizure freedom by study region, the smallest pooled prevalence (27%) of seizure freedom was reported in Tigray region whereas the highest prevalence (52%) was found in Addis Ababa (Figure 3). Regarding the subgroup analysis by age group, the lowest (43%) and the highest (77%) pooled prevalence of seizure freedom was showed in adult epileptic patients and pediatric epileptic patients, respectively (Figure 4). The majority of the studies (73.91%) used seizure freedom for at least a 6-month period to define controlled seizure, and the remaining studies (26.09%) used seizure freedom for less than a 6-month period to define controlled seizure. The pooled prevalence of seizure freedom varied by the duration of seizure-free period, and the higher pooled prevalence of seizure freedom (58%) was found in seizure-free period of less than 6 months (Figure 5).

### 3.5. Factors Associated with Poor Treatment Outcome (Uncontrolled Seizure) among Patients Treated with Antiepileptic Drugs

Among the 23 articles which were included in this systematic review, 15 studies reported only magnitude of seizure freedom and 8 studies (34.78%) [7–9, 13, 17, 28–30] reported both magnitude of seizure freedom and factors associated with uncontrolled seizure. Age at onset of seizure less than 15 years old [13], male gender [7], monotherapy [28], alcohol consumption, negative belief towards medication, presence of comorbidity (29), head injury, sleep deprivation, and exposure to noise and light [17] were reported to be associated with uncontrolled seizure. Although a number of factors were reported as determinant of uncontrolled seizure, variables reported as predictors of uncontrolled seizure among at least two primary studies were considered for this meta-analysis. Therefore, medication nonadherence and frequent seizure before the start of antiepileptic drugs were included in this meta-analysis to measure their crude association with uncontrolled seizure.

**Figure 6:**
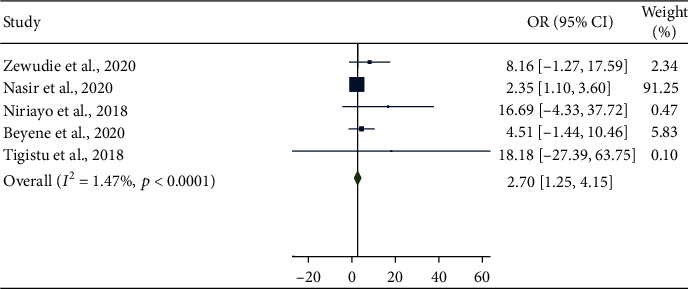
Forest plot depicting pooled random-effect size (OR) of nonadherence.

**Figure 7:**
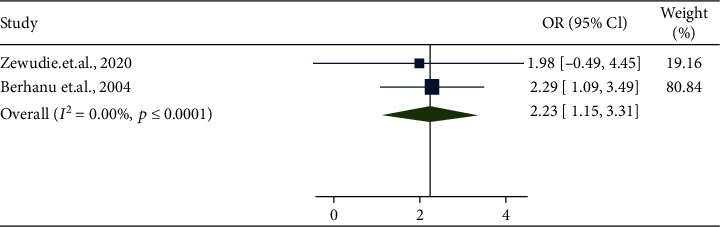
Forest plot depicting pooled random-effect size (OR) of seizure frequency before treatment.

The result of the meta-analysis revealed that medication nonadherence and frequent seizures before the start of antiepileptic drugs were significantly associated with uncontrolled seizure (OR = 2.70, 95% CI: 1.25-4.15 (Figure 6); OR = 2.23, 95% CI: 1.15-3.31 (Figure 7), respectively).

### 3.6. Publication Bias

**Figure 8:**
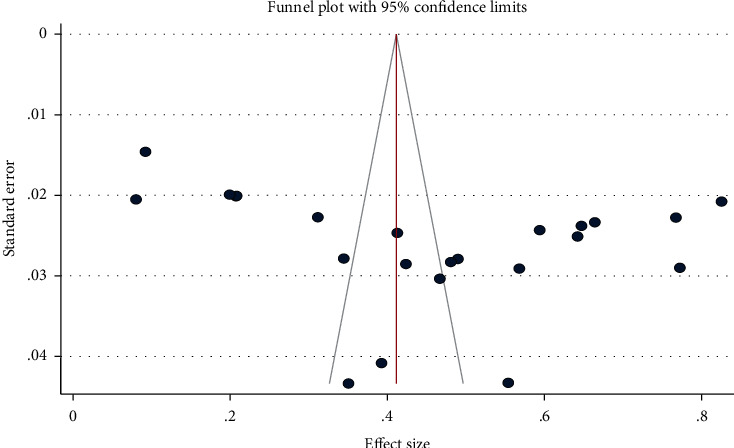
Funnel plot of the prevalence of controlled seizure studies.

The visual inspection from the funnel plot assessment revealed that it is symmetrical and has not showed publication bias. Likewise, Egger's test also revealed that there was no publication bias (Egger's test, *p* = 0.37) (Figure 8).

## 4. Discussion

The present systematic review found that 46% of the epileptic patients experienced seizure freedom following treatment with antiepileptic drugs in Ethiopia. As far as the authors' knowledge, this is the first systematic review and meta-analysis in Ethiopia to find the pooled prevalence of controlled seizure and determinants of uncontrolled seizure. The finding of this systematic review has a great importance to improve the quality of care for people suffering from epilepsy by showing the summarized prevalence of controlled seizure and suggesting possible strategies to improve the level of controlled seizure among people taking antiepileptic drugs. Moreover, the review can have clinical importance and potential policy response for healthcare systems of Ethiopia. Hence, increasing the level of controlled seizure is essential to enable epileptic patients achieve daily activities and to decrease their mortality [1, 32].

In this systematic review, the magnitude of controlled seizure was 46% among patients receiving antiepileptic drugs, which is inconsistent with the Cochrane review, World Health Organization (WHO) report, and other clinical guidelines [1, 33, 34]. The discrepancy may be explained by the variation in the study design, study population, comorbidity, definition of controlled seizure, and level of medication nonadherence of the included primary studies. The reason of uncontrolled seizure in the current systematic review may be due to the presence of pharmacoresistant epilepsy [35, 36] and nonadherence to antiepileptic drugs which was reported by a systematic review in Ethiopia [37]. Therefore, identifying pharmacoresistant epilepsy and taking appropriate management options for it are essential along with increasing medication adherence level of epileptic patients to optimize treatment outcome.

The subgroup analysis of the current systematic review and meta-analysis revealed that there was significant difference between reports of controlled seizure among primary studies based on their difference in study region, seizure-free period, and age group. Accordingly, the highest prevalence was observed in studies conducted at Addis Ababa (52%, 95% CI: 29-56), a study which included only pediatric patients (77%, 95% CI: 71-83), and studies which used seizure-free period of less than six months to define controlled seizure (58%, 95% CI: 32-83). This disagreement can be explained by the fact that pediatric patients tend to be more adhered to their antiepileptic drugs because their parents/caregivers may always advise to take their medication and even parents/caregivers may force the child to take the medication. In addition, the difference in etiology of epilepsy between adult and pediatric patients may be the reason in part for discrepancy of results. There was insufficient number of study in pediatric epileptic patients about seizure control which made difficult to compare it with the findings from adult epileptic patients. The higher prevalence of controlled seizure may be more likely to be found in short period than in long period of follow-up because on long term, the chance of drug resistance, comorbidity, and exposure to factors that trigger seizure or other factors may be increased [17, 29]. Regarding the definition of controlled seizure, in the present review, 17 articles (73.91%) used at least the definition of controlled seizure recommended by the International League Against Epilepsy (ILAE) [38], whereas the remaining papers (26.09%) used less than six-month period to define controlled seizure.

The next objective of the current systematic review was to determine the determinant of uncontrolled seizure among all age group epileptic patients in Ethiopia. The presence of higher seizure frequency before the start of antiepileptic drugs and nonadherence to antiepileptic drugs were significantly associated with uncontrolled seizure. Accordingly, epileptic patients with higher seizure frequency before receiving antiepileptic drugs were 2.70 times more likely to have uncontrolled seizure compared to their counterparts. This may be explained in part by the fact that patients with higher seizure frequency can have a risk of head injury due to convulsion that triggers seizure [17]. It is ascertained that medication nonadherence was responsible for uncontrolled seizure [29, 30, 37]. The result of this meta-analysis also confirmed this conclusion by revealing that nonadherent participants were 2.23 times more likely to have uncontrolled seizure compared to participants which adhered to their medication.

In general, the findings of this systematic review and meta-analysis revealed that almost half of the people attending the outpatient department of epilepsy clinics in Ethiopia had uncontrolled epilepsy which needs a team effort to identify the main determinant factors (such as pharmacoresistant epilepsy and medication nonadherence) and to take appropriate decisions in a routine clinical care. Therefore, professions are recommended to consider the importance of the psychosocial support system and its integration with the pharmacological intervention [39, 40]. Designing strategies to identify pharmacoresistant epilepsy and its treatment and increasing medication adherence are recommended in Ethiopia.

### 4.1. Strength and Limitations of the Study

An extensive search of databases and grey literatures was conducted in this systematic review and meta-analysis. The quality of primary studies which were included in this review was assessed by using standardized measurement, and all articles met the specified criteria. However, the following points can be considered as the limitations of the present study. First, it was not possible to evaluate the role of some of the most important information such as epilepsy's etiology, age, gender, epilepsy length, and numbers of antiepileptic drugs used because this information was lacking in the considered studies. Second, only articles written or translated to English were selected for this systematic review.

## 5. Conclusion

In this systematic review, the prevalence of controlled seizure was found to be low among epileptic patients receiving antiepileptic drugs at the outpatient department of epilepsy clinics in Ethiopia. There was difference in definition of controlled seizure, inclusion of age group, and study regions in the primary studies which showed significant variation in prevalence of controlled seizure.

Higher frequency of seizure before treatment and medication nonadherence had crude association with uncontrolled seizure. This warrants that clinicians should give more focus to epileptic patients regarding monitoring and evaluation of treatment outcome of epilepsy and factors that affect seizure control in routine clinical services.

The use of standardized definition of controlled seizure, designing strategies to identify pharmacoresistant epilepsy and its treatment, and increasing medication adherence are recommended in Ethiopia.

## Figures and Tables

**Table 1:** Summary of primary studies of reporting treatment outcome among all age group epileptic patients in Ethiopia (*n* = 23).

Author	Publication year	Study region	Study design	Sample size	Outcome	Seizure-free in months	Quality score	Prevalence (%)
Zewudie et al. [30]	2020	SNNP	CS	143	56	≥24	8	39.20
Birru et al. [14]	2016	Amhara	CS	336	277	3	9	82.4
Gurshaw et al. [20]	2014	Oromiya	CS	290	164	36	8	56.7
Tefera et al. [22]	2020	Oromiya	CS	121	42	24	9	35
Nasir et al. [28]	2020	Addis Ababa	CS	291	100	12	9	34.4
Tefera et al. [8]	2015	Oromiya	CS	132	146	≥12	8	55.3
Gebre et al. [6]	2018	Tigray	CS	175	14	1	9	8
Mekuriaw et al. [31]	2020	SNNP	CS	321	157	12	7	48.9
Tsegabrhan et al. [21]	2014	Oromiya	CS	300	127	1	8	42.3
Haile et al. [27]	2018	AA	CS	410	272	12	9	66.3
Berhanu et al. [13]	2004	Amhara	CS	312	150	≥12	7	48
Tegegne et al. [26]	2015	AA	CS	415	129	12	9	31.1
Bifftu et al. [15]	2015	Amhara	CS	405	84	12	9	20.7
Bifftu et al. [16]	2017	Amhara	CS	409	85	12	9	20.78
Niriayo et al. [29]	2018	Tigray	CS	270	126	12	8	46.6
Beyene et al. [7]	2020	Amhara	Rcoh	210	162	3	7	77.1
Gidey et al. [9]	2019	Oromiya	Rcoh	404	261	≥12	8	64.6
Tigistu et al. [17]	2018	Amhara	CS	408	242	2	9	59.3
Fanta et al. [25]	2015	AA	CS	346	265	3	7	76.6
Kiflie et al. [19]	2011	Oromiya	CS	365	234	≥6	8	64.1
Tareke et al. [18]	2020	Amhara	CS	398	164	≥6	8	41.2
Abadiga et al. [23]	2019	Oromiya	CS	392	36	12	7	9.2
Engidaw et al. [24]	2020	Oromiya	CS	402	80	12	6	19.2

AA: Addis Ababa; CS: cross-sectional; Rcoh: retrospective cohort; SNNP: Southern Nation, Nationalities, and People.

## Data Availability

The data analyzed and used to prepare this study are available from the corresponding author upon rational request.
